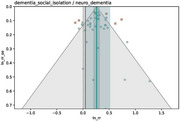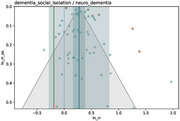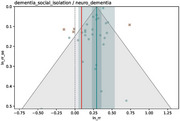# Social Isolation and Risk of Dementia: A Burden of Proof Study

**DOI:** 10.1002/alz70860_103742

**Published:** 2025-12-23

**Authors:** Channa Buxbaum, Natalie Chen, Anh Thy Nguyen, Jamileh Shadid, Kate Gillespie, Damian Santomauro, Liane Ong, Jamie Steinmetz

**Affiliations:** ^1^ Institute for Health Metrics and Evaluation, Seattle, WA, USA; ^2^ University of Queensland, Brisbane, QLD, Australia

## Abstract

**Background:**

Social isolation is increasingly recognized as a public health concern, particularly for older adults. However, existing reviews are often limited in scope or fail to adequately account for heterogeneity across studies. To address these gaps, we conducted a systematic review and meta‐analysis of broader evidence to quantify the relative risk of dementia associated with social isolation, while examining individual isolation measures through subgroup analyses.

**Method:**

We reviewed PubMed, Web of Science, and PsycInfo to identify longitudinal studies published between 1980 and 2024. The Burden of Proof (BoP) methodology, developed for the Global Burden of Disease study, was applied to adjust for systematic biases and incorporate heterogeneity across studies, providing a conservative, robust interpretation of the evidence. We included studies reporting mean relative risks (RR) with 95% uncertainty intervals (UI) for dichotomous measures of social isolation. Primary analyses included all measures of objective social isolation, while subgroup analyses focused on dimensions such as social networks (e.g., small size or low frequency of social connections) and social activities (e.g., group meeting attendance). We additionally conducted an analysis with 13 studies examining loneliness, a related but distinct construct to social isolation.

**Result:**

Of the 1,225 references screened, 41 studies met the inclusion criteria, encompassing a diverse range of social isolation measures. Social isolation revealed a possible association with dementia risk (mean RR: 1.29, 95% UI 0.98–1.71). Subgroup analyses indicated that lack of social activity was associated with increased dementia risk (mean RR: 1.34, 95% UI: 1.05–1.71). Conversely, lack of social network (mean RR: 1.31, 95% UI: 0.76–2.28) did not exhibit a clear association once heterogeneity was fully considered. Loneliness (mean RR: 1.33, 95% UI: 0.69–2.59) likewise did not exhibit a consistent relationship with dementia.

**Conclusion:**

Social isolation is a modifiable risk factor for dementia risk, with lack of social activity emerging as the dimension most consistently associated with risk. Public health interventions should target specific aspects of social isolation demonstrating the greatest impact on dementia risk, while future research should aim to refine measurement methods to enhance comparability across studies.